# Determinants of health care worker breastfeeding experience and practices and their association with provision of care for breastfeeding mothers: a mixed-methods study from Northern Thailand

**DOI:** 10.1186/s13006-024-00613-4

**Published:** 2024-01-25

**Authors:** Mary Ellen Gilder, Chanapat Pateekhum, Nan San Wai, Prapatsorn Misa, Phimthip Sanguanwai, Jarntrah Sappayabanphot, Nan Eh Tho, Wichuda Wiwattanacharoen, Nopakoon Nantsupawat, Ahmar Hashmi, Chaisiri Angkurawaranon, Rose McGready

**Affiliations:** 1 Global Health and Chronic Conditions Research Group, Chiang Mai University, Chiang Mai, Thailand; 2 Shoklo Malaria Research Unit, Mahidol-Oxford Tropical Medicine Research Unit, Faculty of Tropical Medicine, Mahidol University, Mae Sot, Tak, Thailand; 3 Mae Ramat Hospital, Mae Ramat, Tak, Thailand; 4 Mae Tao Clinic, Mae Pa, Tak, Thailand; 5 Saraphi Hospital, Saraphi, Chiang Mai, Thailand; 6 Institute for Implementation Science, University of Texas Health Sciences Center (UTHealth), Houston, TX USA; 7 Department of Health Promotion and Behavioral Sciences, School of Public Health, University of Texas Health Sciences Center (UTHealth), Houston, TX USA; 8 Centre for Tropical Medicine and Global Health, Nuffield Department of Medicine, University of Oxford, Oxford, UK

**Keywords:** Breastfeeding, Midwives, Nurses, Working mothers, Workplace, Maternity protection

## Abstract

**Background:**

Improving breastfeeding rates is one of the most cost-effective ways to prevent infant deaths, but most of the world falls far below WHO recommended breastfeeding practices. Confident, informed healthcare workers are an important resource to promote breastfeeding, but healthcare workers are at risk of early breastfeeding cessation themselves. Culture, ethnicity and socio-economic status impact breastfeeding rates with some of the highest and lowest rates in Southeast Asia reported from Thailand. This study explores the relationship between workplace determinants of breastfeeding, personal breastfeeding outcomes for healthcare workers, and the breastfeeding care healthcare workers provide their patients.

**Methods:**

This study used a sequential exploratory design guided by a conceptual framework based on social ecological/ecological psychology models. Participants came from four clinical sites in Northern Thailand, from ethnically Burman or Karen communities with high breastfeeding rates, and Thai communities with low breastfeeding rates. In-depth interviews (July 2020-November 2020) were followed by a quantitative survey (November 2020-July 2021) derived from validated questionnaires (Australian Breastfeeding Knowledge and Attitudes Questionnaire and the Workplace Breastfeeding Support Scale) with minor local adaptations.

**Results:**

Interviews highlighted the beneficial effects of supportive workplace policies, the importance of physical spaces to facilitate proximity between mothers and infants, and the problem of low milk production. Meeting the WHO recommended practices of exclusive breastfeeding to 6 months or total breastfeeding to 2 years or more was more common in sites with higher levels of breastfeeding support (aOR 7.3, 95%CI 1.8, 29.1 for exclusive breastfeeding). Exclusive breastfeeding was also higher when staff set breastfeeding goals (aOR 4.4, 95%CI 1.7, 11.5). Staff who were able to see their infants during the work day were less likely to terminate breastfeeding because of work (aOR 0.3, 95%CI 0.1, 0.8). Staff who met both WHO recommendations themselves were more likely to report high levels of confidence caring for breastfeeding patients (aOR 2.6, 95%CI 1.1, 6.4).

**Conclusions:**

Workplace protections including supportive maternity leave policies and child-friendly spaces can improve breastfeeding outcomes for healthcare workers. These improved outcomes are then passed on to patients who benefit from healthcare workers who are more confident and attentive to breastfeeding problems.

**Supplementary Information:**

The online version contains supplementary material available at 10.1186/s13006-024-00613-4.

## Background

Breastfeeding prevents infant deaths, especially in the first months of life [1], most dramatically in low-income communities and for infants of women with lower education [1, 2] and is considered to be one of the most cost-effective interventions to save infant lives [3]. The World Health Organization recommends that breastmilk be the exclusive food for infants fewer than six months old and continued as part of the diet of young children up to two years and older [4]. Parental and public health efforts to follow these recommendations are often undermined by the marketing of the multi-billion dollar infant formula industry [5].

Working outside the home is associated with early breastfeeding cessation [6, 7] and healthcare workers (HCW) often have suboptimal breastfeeding outcomes [8–11]. Barriers to breastfeeding for HCW include insufficient time, lack of spaces to breastfeed or express breastmilk, inadequate staffing, short maternity leave, long and unpredictable work hours, and real or perceived low milk production [12]. Workplace policies that support mothers to breastfeed or express breastmilk at work have been shown to increase the amount of breast milk infants receive, in addition to other maternal and infant health benefits [13]. The greatest benefits are seen with comprehensive workplace programs incorporating multiple means of support [13]. The International Labor Organization has codified the right of mothers to maternity leave and protected time for lactation during work hours [14].

Not only do workplace programs supporting breastfeeding help breastfeeding mothers, work policies supporting breastfeeding for HCW may translate to improved patient care. Studies in high income countries have demonstrated a link between physicians’ personal breastfeeding experiences and their ability to support breastfeeding mothers [8, 15]. There is a positive association seen in both high- and middle-income countries between the degree to which HCW encourage and support breastfeeding and patient breastfeeding duration and exclusivity [16, 17]. This “support-experience-care” cascade for non-physician HCW has been explored minimally in resource-constrained settings, often confined to HCW knowledge and attitudes toward breastfeeding [9, 10, 18].

This study was undertaken in two culturally diverse northern Thai provinces and included Royal Thai Government (RTG) hospitals and community-based organization (CBO) clinics. RTG staff are predominantly Thai, while CBO staff are predominantly Karen and Burman (from Myanmar). Thailand has the lowest breastfeeding rates in the region [19]. Breastfeeding initiation and duration both in Myanmar [19] and in displaced communities from Myanmar in Thailand is high [20], but exclusivity is low [21].

This mixed-methods study examines the determinants of HCW breastfeeding experience, duration and exclusivity to understand the role of workplace supports among other influences in these heterogeneous settings. This study also explores the impact of personal breastfeeding experience on the confidence and knowledge HCW have caring for breastfeeding mother/infant dyads.

## Methods

### Study design, participants and setting

**Fig. 1:**
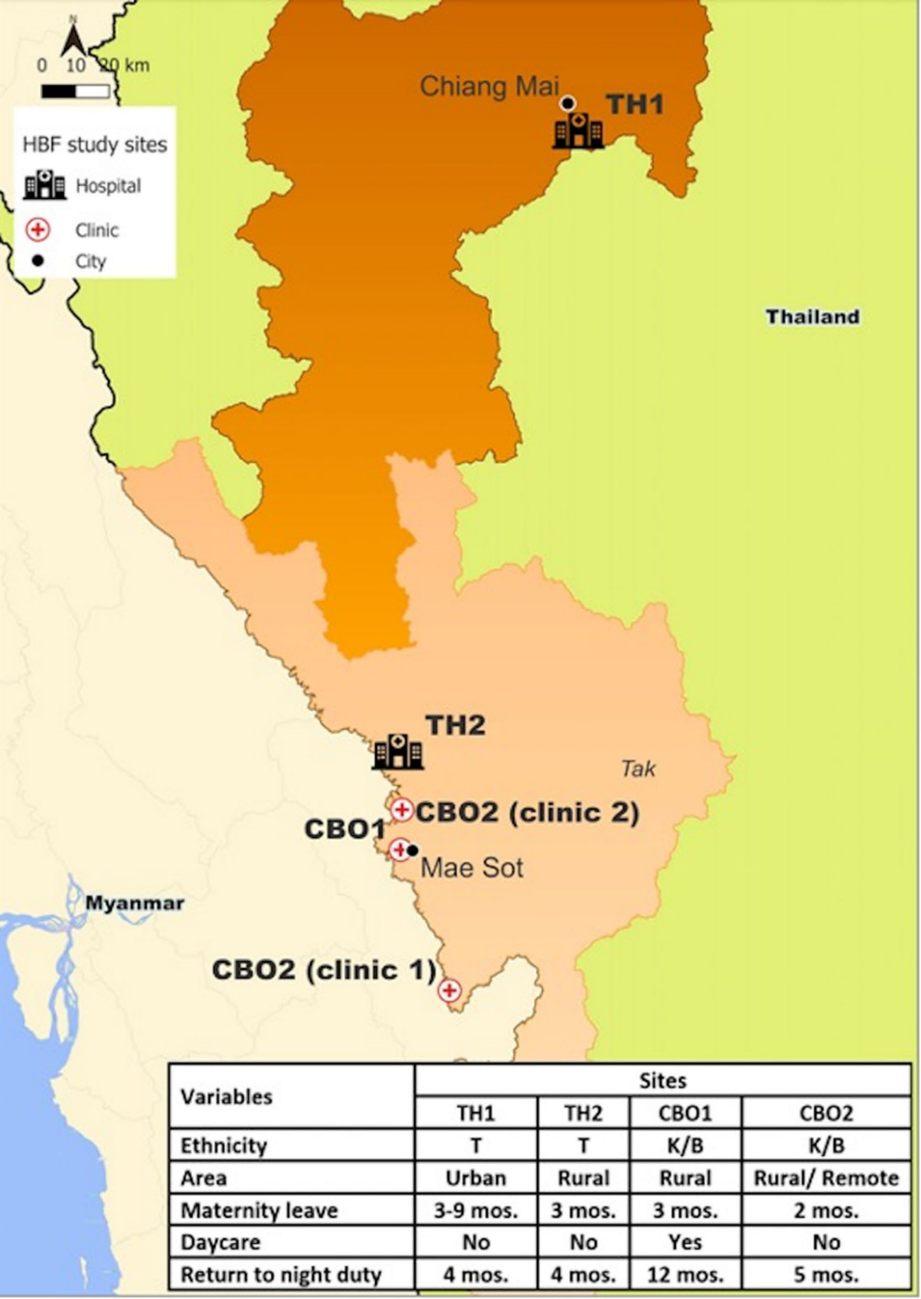
Map of participating healthcare facilities. Site policies around postpartum return to work as well as cultural and setting differences are summarized. Royal Thai Government Hospitals are indicated as “Hospitals”; community-based organization clinics are indicated as “Clinics”. CBO: community based organization; TH: Thai Hospital; T: Thai; K/B: Karen/Burmese; mos: months

We used a sequential exploratory design with in-depth interviews (July 2020 to November 2020) followed by a quantitative survey of breastfeeding experience, knowledge and attitudes of HCW (November 2020 to July 2021). Participants were non-physician HCW from clinics run by two CBOs in Tak Province, Thailand (CBO1 and CBO2), as well as RTG hospitals in and Chiang Mai (TH1) and Tak (TH2) provinces. Staff from CBO2 were working at two separate clinical sites (Fig. 1).

Differences between site settings and policies are described in Fig. 1. CBO HCW are predominantly non-Thai, coming from the estimated 5 million non-Thai workers residing in Thailand [22] and frequently trained by their organization [23, 24]. RTG HCW are predominantly Thai, and formally certified through national training programs. CBO1 was the only site with on-site day care or functional lactation spaces. The cost of daycare was 300 Thai Baht (approximately 10 USD) per month. Electric breast pumps were available to staff at CBO2 since 2019. The RTG hospitals have an official policy that maternity leave can be extended without pay for an additional 6 months, but implementation depends on local administration.

### Conceptual framework and research paradigm

**Fig. 2:**
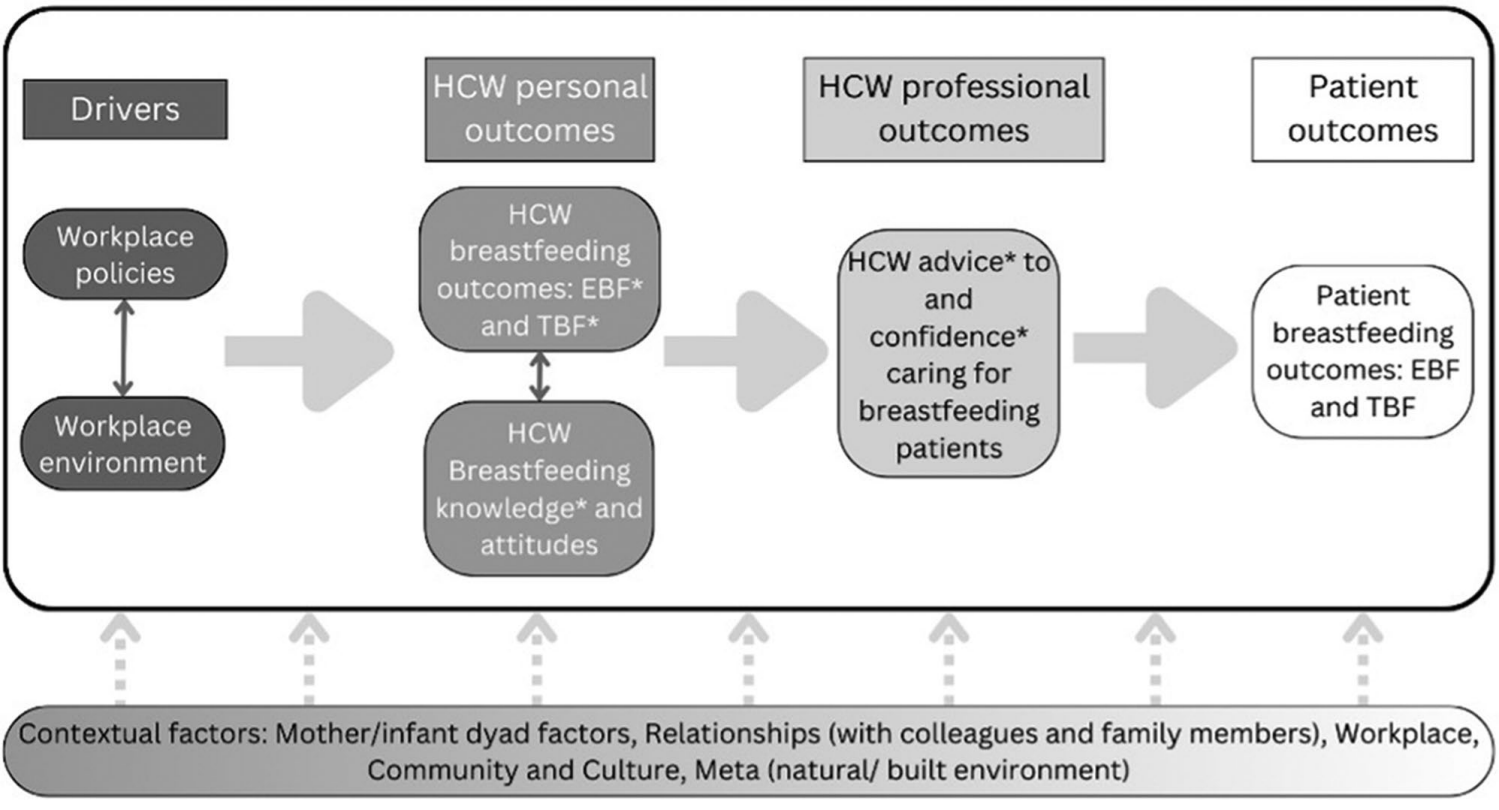
Conceptual Framework. Conceptual framework outlining social ecological/ecological psychology models, contextual factors, and outcomes of interest. Elements of the framework that are study outcomes are indicated with an asterisk (*). Abbreviations: HCW healthcare worker, EBF exclusive breastfeeding, TBF total breastfeeding

At the protocol and tool development stage the social-ecological model, which has previously been applied to breastfeeding [25], was modified for the research context to describe the expanding circles of influence on the central mother/infant dyad, namely: mother/infant dyad factors, relationships (both with family and coworkers), workplace, communities and cultures, and the meta level (which included the natural and built environment, national boundaries, the COVID-19 pandemic, formula advertising etc.). In addition to these larger contextual elements of the social-ecological model, ecological psychology [26] was used to help conceptualize the link between workplace policies, the impact these policies had on personal breastfeeding experience and breastfeeding knowledge among health workers, and how this influenced health worker ability to support breastfeeding patients (Fig. 2). This form of the social-ecological model was chosen because it helped visualize how environment—in this case, the workplace—determines behavior. These concepts were applied to different quantitative and qualitative phenomena throughout the study.

### Qualitative and quantitative study components

#### In-depth interviews (IDI)

A semi-structured interview guide was drafted to elucidate the experience and the impact of the different domains of the social-ecological model on breastfeeding HCW (Fig. 2, Additional File 1). The original interview guide was conceptualized in English, Burmese and Karen by NSW (midwife trainer) and MG (doctor), who conducted all of the Burmese and Karen interviews. The guide was translated from English into Thai (by CP, statistician), and the translation was checked by a bilingual researcher (NN, doctor) who back-translated the guide, confirmed the intended meaning of the questions with MG, and assisted with training of the Chiang Mai interview team (with MG and CP). Tak province Thai interviews were done by PM and JS (social scientists, trained and assisted by MG). As the interviews were semi-structured, interviewers were encouraged to focus on the themes of the interview guide but use flexible wording when asking the questions to maximize participant comfort and demonstrate active listening. Four IDI were planned for each site for a total of 16 interviews, with the option of recruiting up to 24 if saturation (repeated themes within interviews from a site) was not reached. The study team enlisted the help of clinical and human resource (HR) departments to identify interviewees purposively who represented a wide range of experiences including those who did and did not have difficulty breastfeeding. This method of recruitment was used to avoid recruiting only staff with positive experiences or strong feelings about breastfeeding, a likely outcome of open calls for staff to volunteer to join interviews.

Staff conducting IDI were all women experienced with qualitative interview techniques and received specific study training prior to the interviews. Interviews were conducted in a private area with at least two research staff, an interviewer and a note-taker, or by video call if face-to-face meeting was not possible (TH1 only). Interviewers were well known to CBO2 participants, but not participants from other sites. Interviews with RTG staff were conducted in Thai, while interviews with CBO staff were conducted in Karen and/or Burmese according to the interviewee’s preferred language. Following interviews, debriefing between study staff identified and recorded main themes of the interview and sensitive or interesting topics. Interviews lasted 30–60 min, were audio-recorded, translated from the original languages and transcribed in English. Study staff were aware of potential bias from their relationship with CBO2 participants and from the use of HR departments to choose participants. This was mitigated by open interview techniques and triangulation with quantitative data.

Participants were assigned anonymized codes and only study staff and translators had access to the securely stored, password-protected and anonymized interview files.

#### Surveys

An 84-question survey was adapted from validated instruments including the workplace breastfeeding support score (WBSS) [27] and the short form of the Australian Breastfeeding Knowledge and Attitudes Questionnaire (ABKAQ-SF) [15, 28] (Additional File 2). Additional questions were added to ensure all key aspects of the conceptual framework were represented (Fig. 2). Questions included demographics, work experience, workplace supports (maternity leave, respite from night duty, lactation/daycare facilities), birth and breastfeeding experience, reasons for breastfeeding cessation, breastfeeding attitudes, breastfeeding knowledge, and experience and confidence in caring for breastfeeding patients. After translation and back translation, each language version of the survey was piloted with two mothers literate in the respective language who were asked to mark any areas where the wording or options were problematic, and interviewed after taking the survey to ensure understanding about the questions and to clarify problem areas. These notes were brought back to the translators and the corrected version was checked by an additional individual fluent in the relevant language. Residual areas of confusion were brought back to the translator until clarity was achieved, a process requiring multiple iterations in some cases.

Sample size calculations were performed using StataIC 15 [29] based on demonstrating a difference in the proportion of mothers exclusively breastfeeding to 6 months (EBF-6 m) at CBO1 and CBO2 (expected to be the most similar sites). An informal estimate of EBF-6 m at CBO1 was 95% and at CBO2 was 60%, leading to a sample size of 22 HCW mothers per site with 80% power and alpha = 0.05 (Stata command: power twoproportions 0.95 0.6). It was estimated that approximately one-third of HCW would be mothers, and therefore recruitment of approximately 66 total staff at each site would be needed to enroll 22 mothers. Because of uncertainties around the estimates, a goal quota of 75 individuals (including mothers and non-mothers) was used.

Staff working with pregnant or postpartum women, or infants were prioritized and all invited to join the survey. After inviting all high-priority groups, non-physician healthcare workers were invited from other departments to fill the quota without other exclusions (see details in Additional File 3).

### Analysis

#### Qualitative analysis

Anonymized English transcripts were analyzed using Dedoose software [30] using open coding by one researcher (MG). Codes were organized and thematic analysis was performed according to the conceptual framework. At multiple steps throughout this process, codes and themes were confirmed, discussed, and consensus/agreement among research team members was obtained. This led to an internally validated codebook used to code the data. Preliminary thematic analysis was further discussed and confirmed with the research team prior to final thematic analysis following the conceptual model (Fig. 2). Following completion of the study, results were presented back to leadership and staff at the participating organizations for feedback.

#### Quantitative analysis

##### Outcomes

Following the conceptual framework (Fig. 2), some variables (e.g. EBF-6 m) were outcomes for some analyses (e.g. impact of workplace support) and exposures for other analyses (e.g. impact on confidence).

The primary outcomes were based on the WHO recommendations of EBF-6 m and at least two years of total breastfeeding (TBF-2y). EBF-6 m was defined as receiving only breastmilk and no other food or liquids (including water) from birth to 6 month.

with the exception of medications if required [31]. TBF-2y was defined as provision of any breastmilk at the child’s second birthday or after [31]. This could include breastfeeding or feeding expressed breastmilk, in addition to other foods. Survey items determined the primary reason for terminating breastfeeding for the staff’s youngest child. A binary variable for confidence in caring for breastfeeding dyads was derived from a five-point Likert scale, in which responses of 4 or 5 were considered “high”. Staff who reported advising women about EBF-6 m and TBF2y were considered to give correct advice. Scores on 19 breastfeeding knowledge questions (described below) were calculated and a binary variable derived with a cutoff of the 50th centile.

#### Independent variables

Workplaces were categorized as “high breastfeeding support” or “low breastfeeding support” based on qualitative data from in-depth interviews and organizational policies. Sites were further categorized as “high breastfeeding prevalence” or “low breastfeeding prevalence” based on having predominantly Karen/Burmese or Thai staff.

The 11-item Workplace Breastfeeding Support Score (WBSS) was scored as in previous publications [27]. The WBSS is a survey developed and validated in the USA, with questions derived from the existing literature on workplace challenges to breastfeeding, including questions about physical spaces, equipment, and colleague attitudes. The final four questions of the 11-item WBSS were specific to women who had tried to breastfeed or pump at work, and were therefore missing for a significant proportion of women (e.g. those who stopped breastfeeding before returning to work). To account for this, these four questions were omitted in the multivariable regression (which used a WBSS-short form (WBSS-SF)).

The modified ABKAQ-SF was scored as recommended by the original questionnaire developers.(9) Attitude questions used a five-point Likert scale from 1 to 5. Knowledge score (maximum total 19) and attitude score (maximum total 55) were calculated separately. Knowledge questions with correct answers were given one point; wrong or missing answers, and questions marked as “don’t know” were given zero points.

Age, year of child’s birth, and breastfeeding attitude were summarized by quartiles. Quartiles were simplified where appropriate for the final analysis (e.g. lowest age quartile).

All other variables were directly derived from questions in the survey.

#### Statistical analysis

Statistical analysis was done using StataIC 15 [29]. Binary and categorical data were summarized with numbers and proportions. Normally distributed continuous data was summarized with mean and 95% confidence intervals (95% CI) or standard deviation (SD), and non-normal continuous data was summarized using median, interquartile range, and range. Records with data missing on key variables were not included in the analysis.

Survival analysis was used to create a Kaplan-Meier curve [32] to visually compare overall breastfeeding trends at the four sites. In these analyses, cases were censored from the EBF-6 m analysis when they introduced supplemental foods, formula, or water; and from the TBF2y analysis when they stopped breastfeeding.

The logistic regression models were created after univariable analysis using random effects to account for clustering by site. Variables with a p value of < 0.2 on univariable analysis were included in the multivariable model. A p value of < 0.05 was considered statistically significant in the final model. Clustering by *worksite at the time of birth* was controlled for with direct effects in the final analysis of breastfeeding duration and reasons for stopping breastfeeding. Clustering by *current worksite* was controlled for with direct effects in the analysis of confidence, knowledge and advice.

#### Bias

Logistical constraints made probabilistic sampling of the low priority departments at TH1 impossible. To account for this, sensitivity analysis was performed on all of the logistic regression models excluding the TH1 data.

## Results

### In-depth interview results

Seventeen IDI were held with HCW from participating sites (CBO1 4, CBO2 5, TH1 4, TH2 4). The fifth interview was added at CBO2 in order to reach saturation, as its two clinic locations added additional nuance. Participants came from diverse fields from nursing and midwifery to dental and pharmacy, had a range of 1 to 3 children, and breastfeeding duration of the most recent child ranged from 3 months to 3 years.

Themes from IDI mapped to the conceptual framework as summarized in Table 1; Fig. 2. In the workplace, responsive implementation of workplace policies (Theme 1) was an important facilitator of breastfeeding success, with critical supports differing for different women (Theme 2). The local concept of *arr-nar/kreng-jai* (Burmese/Thai words for a sense that one is imposing on or bothering others, Theme 3) [33, 34] was prevalent in the workplace environment. *Arr-nar/kreng-jai* discouraged mothers from taking needed breaks from work to breastfeed or express breastmilk, regardless of official policies and positive relationships with co-workers. When HCW discussed their own breastfeeding outcomes, breastmilk production (Theme 5) and mother-infant bonding (Theme 6) were frequently discussed, both of which were threatened by separation of mother and baby due to work. Personal experience of breastfeeding was universally credited with improved confidence and skills caring for breastfeeding dyads professionally (Theme 7). Family support was essential to breastfeeding success and maternal wellbeing, and was most frequently discussed when support was absent (Theme 8). Culture (Theme 9) was a powerful behind-the-scenes force which participants discussed in the context of patients but not themselves. The practice of giving neonates water was identified as an impediment to exclusive breastfeeding in all local cultures, and Karen/Burman communities were recognized for longer duration and higher prevalence of breastfeeding. Finally, elements of the physical environment (Theme 10)– from rivers to day-care facilities– influenced women’s ability to stay close to their infants. Proximity to their infants was a major determinant of the degree of peace of mind that working mothers experienced.

**Table 1:** – Summary of qualitative themes with exemplary quotes

Framework level	Theme	Quotation
Workplace drivers	1) Responsive implementation of policies	*Actually, I was lucky that the hospital understood me because I felt that 3 months were not enough. I was lucky that my… colleagues understood me, and I was allowed to extend my leave. It helped, I returned to work after passing the difficult period. When I came to work, it was good that my colleagues understand and let me have time for pumping. These things are necessary because it may be difficult if some colleagues don’t understand.* **TH1**
	2) Organizational support	*For me I just have one funny idea– it would be nice to have a clinic for mothers who are breastfeeding because every mother who is breastfeeding has different challenges– it is not all the same. It would be great for mothers to have a training course. So for the mothers who are determined to breastfeed their children but face challenges they could receive advice and support.* **TH2**
	3) Arr-nar/kreng-jai (“not wanting to impose”)	*I would say [maternity leave should be] 6 months, but I feel that it is too long and feel bad for our colleagues. 5 months and a half will do, just a little bit longer.* **TH1**
	4) Organizational leadership	*Working here it feels like a family, looking after each other.* **CBO1**
HCW personal outcomes	5) Breast milk production	*You need to be very consistent with pumping milk because if not consistent, you will not produce as much milk as you need, and since you don’t have a baby to directly breastfeed to stimulate your breast milk production, your production will gradually go down… especially when you don’t have your baby nearby to directly breastfeed. When our baby is directly breastfeeding it induces the release of oxytocin which causes more milk to be produced. If there is no stimulation, and the brain is not triggered, eventually there will be no milk production.* **TH2**
	6) Mother-infant Bonding	*After delivery the mother’s hormones can contribute to stress for the mother. Some patients will have depression from the hormones. When we let our child breastfeed we feel warmth in our heart, it will heal the heart. I also experienced quite often the feeling that I just suddenly want to cry– it is the hormonal effect. When I pick up my child and breastfeed that feeling disappears.* **TH2**
HCW professional outcomes	7) Improved care for breastfeeding mothers	*I can counsel better after I have babies. When I was single, I am not quite good at that. After breastfeeding my two babies, we can share our experiences. We can tell them how to hold the baby.* **CBO1** *They ask me about herbal supplements and ways to increase milk production. I tell them that banana blossoms, ginger tea, and Kaeng Liang (Thai spicy mixed vegetable soup) can stimulate milk production, but also tell them that they can take some medicines to stimulate milk production… I had experienced the same problem. I had a low milk production when I had my first baby.… I sympathize with them because I was there before, so I want to help them. And I’m glad to help.* **TH1**
Contextual factors	8) Family Support	*I would give this suggestion: find one person who can take care of the baby well. A husband who is not using [alcohol or drugs], who loves babies and can take care of them well. When it is time for breastfeeding, he will give the baby to you to breastfeed. When the baby is finished breast feeding, he will take the baby back. For the baby, breastfeeding is important. If breastfeeding is regular and on time, your baby will grow beautiful…* **CBO2**
	9) Culture and traditions	*I live close to a Burman community in my area. They are also very experienced about breastfeeding…. The hilltribe women [breastfeed] more than 2 years. So I can see even 2–3 years they can still breastfeed their children. [For hospital staff] I don’t really know how long because I didn’t ask them. But for some people around 6 and a half months.* **TH2** *We disagree whether we should feed the baby with water following milk for the first 6 months. I don’t think so, but my babysitter, grandma, says it’s a must…* **TH1**
	10) Meta: Proximity and peace/ Separation and stress	*I worried that people will not look after my baby really well. I worry that mosquitos would bite my baby while he was sleeping. Sometimes, I would bring a hammock and let him sleep here [at the clinic]. I felt more peace of mind when I did that.* **CBO2** *That’s why this year if they don’t transfer me [to the city where my children live], I will resign… If I take care of my kids by myself, I feel more peace of mind.* **TH2**

Abbreviations: CBO community based organization, TH Thai Hospital

### Workplace policies

#### Responsive implementation of policies

Written policies about breastfeeding or the postpartum period beyond maternity leave were scarce at the CBO facilities (confirmed through IDI, discussions with HR staff, and review of staff handbook when available). Participants described rules that were negotiated and re-negotiated over the years. (Theme 1)

Negotiation at CBO1 often happened on a case-by-case basis, and two out of the four interviewees from CBO1 had special arrangements made by the administration to resolve problems in childcare related to their clinic duties. These women described feeling like the administration cared for them “like family”.

*The leaders helped me to move to another department so I am okay now. I really appreciate that. [Laughing] They understand me and look out for mothers who are like me… It is really good for us how our organization helps us… They look after us and think for mother and baby.***CBO1**.

Negotiation at CBO2 was done at the department level, using meetings and consensus decisions.

*There was no rule for when to start night shift after delivery, so we got some complaints from our staff… We called a meeting to decide when breastfeeding mothers should start night shift. We agreed on 5 months.***CBO2**.

Occasionally, changes were communicated from the central administration, and mothers resorted to informal agreements with their direct supervisors in order to reach their breastfeeding goals.

*[HR] came and held a meeting and said, “Staff cannot bring children to the clinic at all.” Aaack. I felt very sad…. What can I do? I want to breastfeed… I said, (whispering) “Doctor, I will go back to breastfeed.” If the doctor allows, I would go back by bicycle quick quick quick and back again… I had to do that for 2 months. I couldn’t do that any longer… What could I do? Her grandmother came. If the baby was hungry she brought her to me and I gave breastfeeding in the back of the room.***CBO2**.

RTG Hospital employees worked under the same maternity policies but the implementation differed between the two sites. Half of the TH1 interviewees reported obtaining permission for extended maternity leave. In contrast, a TH2 interviewee reported trying to negotiate for additional leave after 3 months and being denied. Others said they did not consider asking for prolonged leave because it was “not done” at TH2.

*I could take only 3 months’ leave. Actually I wanted to take leave for 6 months. They told me I could take 6 months’ leave according to the new policy, but my supervisor did not allow me to take leave. My supervisor said there were not enough people.***TH2**.

These differences in policies and their implementation had a direct effect on breastfeeding outcomes across both RTG and CBO settings. All four staff who were given accommodations to support breastfeeding were able to EBF-6 m and had positive breastfeeding experiences. Neither of the staff who described unsupportive supervisors reached their breastfeeding goals.

Six months maternity leave was suggested by multiple participants to match the period of exclusive breast feeding recommended by the WHO, and multiple staff expressed that night duty was particularly challenging for breastfeeding mothers.

#### Organizational support

Several interviewees described their workplace as supporting breastfeeding, but without practical mechanisms in place to help mothers, this “support” was experienced as pressure:

*Yes, the hospital supports breastfeeding a lot. They want the children to get breastmilk… The first few days after I delivered this baby, I didn’t have any breastmilk. The first day, not even one drop of milk came out. It was a challenge for me because my husband started to feel sorry for the child and he wanted to go buy formula for the child. But because I am staff in the hospital the seniors wanted me to be a good example for the other people so I tried. So on the third day I even went to ask for medications to increase the milk production.***TH2**.

RTG staff suggested that helpful support would include mechanisms to get help for breastfeeding problems and share knowledge between mothers (Theme 2).

*The Health Promotion Unit organized trainings on how to feed with breastmilk. I would like to participate when I have the opportunity. There were experiences from the experienced mothers shared to new mothers. We learned from them how to cope with problems.***TH1**.

CBO staff requests for organizational support focused on safe spaces for children at the clinics, and financial support to offset the burdensome cost of formula for mothers with (perceived or real) low milk production.

### Workplace environment

#### Not wanting to impose: arr-nar/kreng-jai

In many Southeast Asian cultures, including Burman, Thai and Karen, the concept of imposing on others– *arr-*nar or *kreng-jai–* is powerful. Feelings of *arr-nar/kreng-jai* towards their colleagues were brought up by participants at all the facilities, but were mentioned least at TH1. This manifested as a tension between participants’ physical need to breastfeed or express breastmilk, and cultural pressure not to burden others, particularly supervisors or seniors. Interviewees at TH2 and especially CBO2 described trying to finish their work before going to care for their baby, whereas at CBO1 two mothers described finishing care for their baby first, and then attending to their work (Theme 3).

*[At 6 months] I didn’t try to express breast milk anymore. For the past 4 months I felt I had imposed on my colleagues. Now my baby is 6 months and he can eat. I can give formula. Since the baby was little I disturbed my colleagues. I feel I imposed on my colleagues. My colleagues might complain. I feel I took advantage of my colleagues. I don’t want to impose on my colleagues.***CBO2**.

*On busy days I feel* kreng-jai *and try to finish as much as I can, and then I ask my colleagues if I can finish the rest after I come back. I planned to pump every 3 h but sometimes I couldn’t. I would feel engorgement but it was relieved when I could pump. And when I came home and my baby nursed, it was much better.***TH2**.

Feelings of *arr-nar/kreng-jai* contributed to decreased breastfeeding duration and exclusivity by creating an environment where women were uncomfortable taking breaks for breast milk expression, contributing to decreases in breast milk production and decisions to give formula.

### HCW personal breastfeeding outcomes

#### Breastmilk production

The most commonly mentioned breastfeeding problem was perceived or real insufficient breastmilk (both delayed lactogenesis and underproduction of breastmilk, Theme 5). However, other breastfeeding issues such as overproduction, mastitis, rejection of breast after bottle and vice versa, and other special situations such as breastfeeding twins and HIV were also mentioned.

Underproduction of breastmilk was discussed equally between the different sites. Increasing fluid intake was the initial suggestion by most staff at all sites to increase breastmilk production, followed by herbal supplements and frequent breast emptying. RTG staff commonly described using electric breast pumps and more “scientific” explanations of regulation of milk production through hormonal signals. CBO staff did not describe a mechanism by which frequent breast emptying caused increased milk production, but recognized that frequent feeding usually led to adequate milk production. They rarely mentioned electric breast pumps or scheduled expression of breast milk, but described hand expression when direct breastfeeding was not possible, usually when their breasts were already engorged.

The difficulty finding time to empty breasts at work regularly was clearly identified as the reason for decreased milk production, formula supplementation, and early breastfeeding cessation by several RTG staff, whereas CBO staff struggling with low milk production seemed to have no way to explain it.

*I could only pump 3 times a day, lunch time, evening time, and night time. Yes, so the milk starts to decrease… I think if I took 6 months of leave and my child always directly breastfeed, I might have milk.***TH2**.

*Sometimes there is just not enough breast milk.***CBO2**.

Both the RTG and CBO staff expressed that being with the baby continuously and directly breastfeeding frequently would prevent many of the challenges they experienced as working mothers.

*For my friends in the village who don’t go to work, they just easily breast feed, just fine. But for my working friends, I think it will be a bit worse, they will all be a bit sad.***CBO2**.

### HCW professional outcomes

#### Improved care for breastfeeding mothers

All the interviewees expressed perceived increased competence caring for breastfeeding dyads after breastfeeding their own babies (Theme 7).

*It is helpful because I have a child so I can relate more in detail to them, and I can use my experience to talk to them, compared with someone who doesn’t have children. Those who don’t have children will use whatever they have learned and seen, but since I have a child and I have experience raising a child, I use my experience in addition to what I have learned. And I think it helps a lot.***TH2**.

However, there was a tendency for those who described less successful breastfeeding experiences to give less appropriate advice. Two CBO mothers who ended up supplementing with formula focused on formula supplementation as the main assistance that their organizations could give breastfeeding dyads who were struggling.

### Contextual factors

#### Proximity and peace of mind

The desire of mothers to be close to their infants was mentioned by women from all sites, and women described peace of mind when this was possible (Theme 10).

*There were problems, but when I see my baby’s face I feel happy.***CBO1**.

Elements of the physical space that contributed to women’s experiences included proximity of their home to their work place, availability of physical space to feed their babies or express breast milk at work, and extreme separation from their infants in some cases. Mothers using daycare (at CBO1) or living very close to their workplaces typically described fewer barriers to breastfeeding, especially when proximity was coupled with strong family support.

*In the beginning there were the challenges I mentioned before. But I was able to adjust to it… and my home is near my work. In the beginning I forgot the pumping equipment often so I would need to drive home to get it, but my house was not very far from work. It is around 5–6 min if I drive fast.***TH2**.

CBO2 and TH2 participants talked 3–4 times more frequently than staff at the other sites about a space where they could safely keep and visit their child at the clinic.

*If we could have a room it would be more comfortable to bring a nanny. But now we have to use the corner here and there to do it. There is no designated place… a nursing room would be very helpful for us, it would be enough. I do not need anything special. A normal fan would be nice. A room with windows to get sunlight and some fresh air.***CBO2**.

Two out of the four TH2 participants and one out of the five CBO2 interviewees sent their infants to stay with distant relatives because other solutions to child care were unaffordable or unavailable. In these cases, mothers and infants were separated for weeks or even months at a time, and breastfeeding was invariably terminated.

*I had a problem because there was no one to watch my baby. There was nothing I could do, I had to send my daughter to my mother-in-law. My baby was just 6 months old, I felt so sorry for her… I wanted to keep her with me longer, but there was nobody to take care of her… If her own mother can care for her, her mother will feel more at ease. But I cannot– I have to leave her.***CBO2**.

### Survey results

Overall, 312 eligible participants participated in the survey, representing 78% of participants invited (Additional File 3). As expected, CBO health workers were predominantly born in Myanmar, took the survey in Burmese or Karen, and had their professional training outside the formal Thai system, through NGO, CBO, or Ethnic Health Organization training programs. The RTG staff were almost exclusively born in Thailand, all did the survey in Thai, and received their training through Thai formal health education programs. Baseline characteristics for all participating staff are summarized in Table 2.

**Table 2:** Baseline characteristics for all survey participants – Data are presented as n (%) unless otherwise indicated. Worksite refers to worksite at the time of the survey

Characteristic			Overall	CBO1 (hi/hi)*	CBO2 (hi/lo)	TH1 (lo/hi)	TH2 (lo/lo)
No children			114/311 (37)	25/74 (34)	40/89 (45)	19/75 (25)	30/73 (41)
Mothers			169/311 (54)	37/74 (50)	34/89 (38)	56/75 (75)	42/73 (58)
Fathers			28/311 (9)	12/74 (16)	15/89 (17)	0/75 (0)	1/73 (1)
Were breastfed themselves	no/don’t know		17/309 (5)	0/74 (0)	2/89 (2)	6/73 (8)	9/73 (12)
	yes		292/309 (95)	74/74 (100)	87/89 (98)	67/73 (92)	64/73 (88)
Years working			10 [5.5–15.6] (0-39.4)	10 [6.25-13] (1–30)	10 [6–13] (0–27)	20 [7.7–25.6] (1-39.4)	9 [4–18] (0.4–38)
Formal training			132/312 (42)	1/75 (1)	4/89 (4)	64/75 (85)	63/73 (86)
Completed high school			244/312 (78)	49/75 (65)	56/89 (63)	71/75 (95)	68.73 (93)
Patient exposure > 10/3mo			199/312 (64)	38/75 (51)	65/89 (73)	44/75 (59)	52/73 (71)
BF attitudes (quartiles from least favorable to most favorable)		1	83/311 (27)	32/75 (43)	30/89 (34)	12/74 (16)	9/73 (12)
		2	76/311 (24)	19/75 (25)	26/89 (29)	17/74 (23)	14/73 (19)
		3	78/311 (25)	14/75 (19)	24/89 (27)	19/74 (26)	21/73 (29)
		4	74/311 (24)	10/75 (13)	9/89 (10)	26/74 (35)	29/73 (40)
High Confidence (4–5)			138/287 (48)	38/74 (51)	38/84 (45)	29/58 (50)	33/71 (46)
Knowledge score: mean % correct (sd)			34.7 (15.5)	32.5 (12.9)	28.8 (9.9)	49.9 (20.1)	31.9 (10.9)
Correct advice			143/288 (50)	39/73 (53)	29/84 (35)	27/59 (46)	48/72 (67)

* Sites are categorized as high “hi” or low “lo” breastfeeding prevalence/organizational support based on the IDI data e.g. hi/lo refers to “high breastfeeding prevalence with low organizational support”

**Fig. 3:**
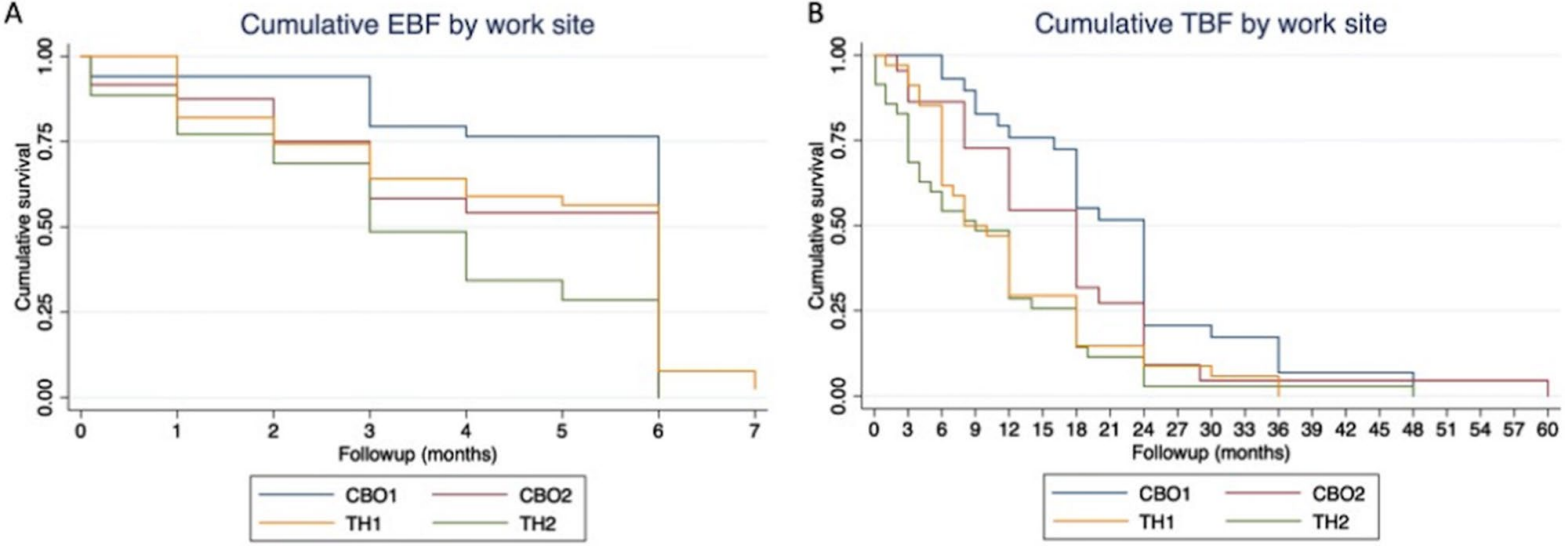
Survival curves for exclusive (**A**) and total breastfeeding (**B**) by site. Exclusive and total breastfeeding duration is visualized by site using Kaplan-Meier curves. Abbreviations: EBF exclusive breastfeeding, CBO community based organization, TH Thai hospital, TBF total breastfeeding

Among the facilities in this study, breastfeeding rates were highest at CBO1 by all measures, and lowest at TH2. In general, breastfeeding duration and exclusivity was highest in the Burman/Karen culture-dominant CBO clinics, and lower in the Thai culture-dominant RTG Hospitals, but significant variation was apparent within each of these categories (Fig. 3). When work sites were categorized as “high breastfeeding support” (CBO1 and TH1) or “low breastfeeding support” (CBO2 and TH2) based on the qualitative analysis, high breastfeeding support compensated for a lower community breastfeeding prevalence at TH1, resulting in comparable breastfeeding outcomes at 6 months at TH1 and CBO2.

#### WHO recommended breastfeeding practices

Overall, 53% of mothers who participated in the survey reported EBF-6 m, 31% reported TBF2y, and 21% met both goals. Detailed characteristics and outcomes for mothers are found in Table 3. CBO1 had the highest breastfeeding rates with 46% of CBO1 staff reaching both WHO recommendations.

**Table 3:** Baseline characteristics and outcomes for mothers – Summary statistics are provided for mothers who completed the survey. Worksite for mothers refers to worksite at the time of birth

Characteristic			Overall	CBO1 (hi/hi)*	CBO2 (hi/lo)	TH1 (lo/hi)	TH2 (lo/lo)
Mother/ infant dyad	Age of mother at delivery†	18–27	39/131 (30)	11/33 (33)	7/24 (29)	8/39 (21)	13/35 (37)
		28–43	92/131 (70)	22/33 (67)	17/24 (71)	31/39 (79)	22/35 (63)
	Baby rejected breast after bottle		39/131 (30)	10/33 (30)	8/24 (33)	11/39 (28)	10/35 (29)
	Set goals for breastfeeding duration		89/131 (68)	28/33 (85)	19/24 (79)	23/39 (59)	19/35 (54)
	Positive BF experience		120/131 (92)	31/33 (94)	24/24 (100)	36/39 (92)	29/35 (83)
	Cesarean birth		50/131 (38)	9/33 (27)	8/24 (33)	14/39 (36)	19/35 (54)
	Hospital birth (vs. clinic or home)*		100/131 (76)	13/33 (39)	13/24 (54)	39/39 (100)	35/35 (100)
	Stopped BF because not enough milk		40/131 (31)	3/33 (9)	2/24 (8)	19/39 (49)	16/35 (46)
	Stopped BF because of work		29/131 (22)	4/33 (12)	10/24 (42)	9/39 (23)	6/35 (17)
Worksite	WBSS *n* = 152		41.4 (7.0)	39.5 (6.2)	35.4 (5.6)	42.8 (6.3)	45.8 (6.0)
	WBSS-SF		24.9 (5.4)	24.0 (4.3)	20.6 (4.1)	25.2 (5.8)	28.6 (4.3)
	Maternity leave (ML) (months)		3 [3–3] (0–10)	3 [3–3] (0.5-3)	2 [2–2] (1–3)	3 [3–3] (2–10)	3 [3–3] (0–8)
	Felt ML was adequate		90/130 (69)	26/33 (79)	14/24 (58)	28/38 (74)	22/35 (63)
	Had night duty		65/130 (50)	13/33 (39)	15/24 (63)	19/38 (50)	18/35 (51)
	Used Day care *n* = 153		48/131 (37)	27/33 (82)	13/24 (54)	1/39 (3)	7/35 (20)
	Expressed breastmilk at work		88/131 (67)	24/33 (73)	14/24 (58)	27/39 (69)	23/35 (66)
	Breastfeeding is common at work		96/131 (73)	27/33 (82)	14/24 (58)	24/39 (79)	24/35 (69)
	Baby close, can visit during work		84/130 (65)	29/33 (88)	17/24 (71)	14/38 (37)	24/35 (69)
	Baby sent to distant relatives		22/130 (17)	1/33 (3)	3/24 (13)	13/38 (34)	5/35 (14)
Year of Birth	1990–2009		33/131 (25)	3/33 (9)	2/24 (8)	14/39 (36)	14/35 (40)
	2010–2015		36/131 (27)	12/33 (36)	7/24 (29)	12/39 (31)	5/35 (14)
	2016–2017		27/131 (21)	6/33 (18)	8/24 (33)	5/39 (13)	8/35 (23)
	2018–2021		35/131 (27)	12/33 (36)	7/24 (29)	8/39 (21)	8/35 (23)
WHO goals	Exclusive breastfeeding 6 months		69/130 (53)	25/33 (76)	13/24 (54)	21/38 (55)	10/35 (29)
	Total breastfeeding ≥ 2 years		40/130 (31)	19/33 (58)	8/24 (33)	9/38 (24)	4/35 (11)
	Both		27/129 (21)	15/33 (45)	5/24 (26)	5/37 (14)	2/35 (6)

Abbreviations: BF breastfeeding, WBSS Workplace Breastfeeding Support Scale, -SF -short form, ML maternity leave, sd standard deviation

† < 27 years was the lowest quartile of ages at the birth of the most recent child

* Sites are categorized as high “hi” or low “lo” breastfeeding prevalence/organizational support based on the IDI data e.g. hi/lo refers to “high breastfeeding prevalence with low organizational support”

Factors associated with reaching the WHO recommendations of EBF-6 m and TBF2y are presented in Table 4; Fig. 3. The strongest predictor of EBF-6 m was working at a site with high levels of breastfeeding support: CBO1 (adjusted odds ratio (aOR) 7.3 95%CI 1.8–29.1, p 0.005) or TH1 (aOR 6.3 95%CI 1.8–21.6, p 0.003). EBF-6 m was significantly more common for mothers in the youngest age quartile (< 28 years old, aOR 3.5 95%CI 1.2–9.6, p 0.018), and mothers who set breastfeeding goals (aOR 4.4, 95%CI 1.7–11.5, p 0.002).

**Table 4:** Characteristics associated with staff meeting WHO exclusive or total breastfeeding goals. The results of univariable and multivariable logistic regression analysis of relationships between work site and personal factors and attaining the WHO exclusive and total breastfeeding recommendations are presented

			Exclusive BF to 6 months (*N* = 130)				Breastfeeding to 2 years (*N* = 130)			
			univariable		Multivariable*		univariable		Multivariable**	
**Characteristic**			proportion (%)	p	aOR (95%CI)	p	proportion (%)	p	aOR (95%CI)	p
Work site	Low BF prevalence, low support (TH2)		10/35 (29)	ref	-		4/35 (11)	ref	-	-
	Low BF prevalence, high support (TH1)		**21/38 (55)**	**0.023**	**6.3 (1.8–21.6)**	**0.003**	9/38 (24)	0.180	3.7 (0.9–15.0)	0.068
	High BF prevalence, low support (CBO2)		13/24 (54)	0.051	2.7 (0.6–13.1)	0.182	**8/24 (33)**	**0.048**	3.6 (0.8–16.3)	0.094
	High BF prevalence, high support (CBO1)		**25/33 (76)**	**< 0.001**	**7.3 (1.8–29.1)**	**0.005**	**19/33 (58)**	**< 0.001**	**6.3 (1.6–24.6)**	**0.008**
	Reason for stopping breastfeeding	not enough milk	19/39 (49)	0.704	1.5 (0.5–4.6)	0.472	**5/40 (13)**	**0.004**	**0.2 (0.1–0.7)**	**0.012**
		work	12/29 (41)	0.142	0.8 (0.3–2.5)	0.715	**5/29 (17)**	**0.011**	**0.3 (0.1–0.9)**	**0.028**
		other/still BF	38/62 (61)	ref			**30/61 (49)**	ref		
	Expressed breastmilk at work	no	16/43 (37)	ref			9/42 (21)	ref		
		yes	**53/87 (61)**	**0.013**	1.6 (0.6–4.3)	0.359	31/88 (35)	0.124	1.6 (0.5–4.4)	0.404
	Used Day care^#^	no	36/82 (44)	ref			17/82 (21)	ref		
		yes	**33/48 (69)**	**0.045**			23/48 (48)	0.061		
Mother/ infant dyad	Age at delivery†	< 28 years	**26/39 (66)**	**0.024**	**3.5 (1.2–9.6)**	**0.018**	6/24 (25)	0.666		
		≥ 28 years	**43/91 (47)**	**ref**			34/106 (32)	ref		
	Place of birth^#^	clinic/home	22/31 (71)	ref			**18/31 (58)**	ref		
		hospital	47/99 (47)	0.242			**22/99 (22)**	**0.018**		
	Set a goal	no	**11/41 (27)**	**ref**			9/41 (22)	ref		
		yes	**58/89 (65)**	**< 0.001**	**4.4 (1.7–11.5)**	**0.002**	31/89 (35)	0.492		
	Finished High school	no	**20/27 (74)**	**0.031**	3.0 (0.8–10.5)	0.089	12/27 (44)	0.457		
		yes	**49/103 (48)**	**ref**			28/103 (27)	ref		
	Baby rejected the breast after bottle	no	53/91 (58)	ref			30/91 (33)	ref		
		yes	16/39 (41)	0.056	0.4 (0.2–1.1)	0.071	10/39 (26)	0.350		

All results in bold are those that are statistically significant (p-value < 0.05)

Abbreviations: BF, breastfeeding; ref, reference

^#^ omitted from multivariable analysis because of collinearity with work site

† < 27 years was the lowest quartile of ages at the birth of the most recent child

* adjusted for work site, reason for stopping breastfeeding, breast milk expression at work, day care use, goal setting, high school completion, breast rejection after bottle use, and year of birth (not significant)

** adjusted for work site, reason for stopping breastfeeding, breast milk expression at work, and year of birth (not significant)

TBF2y was less strongly associated with identified variables. The aOR of TBF2y was at least 3 times higher for the sites with high breastfeeding prevalence or support compared with TH2 (low breastfeeding prevalence and low support), but this was only significant at CBO1 (high breastfeeding prevalence and high support, aOR 6.3, 95%CI 1.6–24.6, p 0.008). When community breastfeeding prevalence, rather than site, was used in the multivariable analysis, Karen/ Burman community members were twice as likely to breastfeed to 2 years (aOR 2.6, 95%CI 1.0-6.6, p0.054). In addition, mothers were less likely to TBF2y if their reason for stopping breastfeeding was low milk production (aOR 0.2, 95%CI 0.1–0.7, p 0.012) or difficulties continuing breastfeeding while working (aOR 0.3, 95%CI 0.1–0.9, p 0.028).

Meeting both WHO goals (Additional File 4) was associated with working at CBO1 (high breastfeeding prevalence and high support, aOR 8.3, 95%CI 1.4–48.4, p 0.018), setting breastfeeding goals (aOR 5.5, 95%CI 1.1–27.3, p 0.029), and having the most recent child after 2015 (aOR 3.3, 95%CI 1.2–9.5, p 0.030).

#### Reasons for breastfeeding cessation: work and low milk production

Low milk production (perceived or real) was the most common reason cited for breastfeeding cessation (46/169, 27%, details in Tables 3 and 5) and was strongly associated with a negative experience of breastfeeding (aOR 9.2, 95%CI 1.2–71.9, p 0.034). Stopping breastfeeding due to insufficient milk production was less common among staff working for a CBO: CBO1 (high breastfeeding prevalence and high support, aOR 0.1, 95%CI 0.0-0.5, p 0.005) or CBO2 (high breastfeeding prevalence and low support, aOR 0.2, 95%CI 0.0-0.9, p 0.040).

**Table 5:** Characteristics associated with attributing the decision to stop breastfeeding to low milk production or difficulties faced breastfeeding while working. The results of univariable and multivariable logistic regression analysis of relationships between work site and personal factors and reasons for cessation of breastfeeding are presented

			Insufficient breastmilk				Difficult to breastfeed and work			
			Univariable (clustered)		Multivariable*		Univariable (clustered)		Multivariable	
**Characteristic**			proportion (%)	p	aOR (95%CI)	p	proportion (%)	p	aOR (95%CI)	p
Work Site	Low BF prevalence, low support (TH2)		16/35 (46)	ref			9/39 (23)	ref		
	Low BF prevalence, high support (TH1)		10/39 (49)	0.796	1.3 (0.5–3.8)	0.610	6/33 (18)	0.611	1.1 (0.3–4.2)	0.935
	High BF prevalence, low support (CBO2)		**2/24 (8)**	**0.006**	**0.2 (0.0-0.9)**	**0.040**	10/24 (42)	0.057	3.1 (0.7–14.9)	0.148
	High BF prevalence, high support (CBO1)		**3/33 (9)**	**0.002**	**0.1 (0.0-0.5)**	**0.005**	4/33 (12)	0.495	0.7 (0.1–3.7)	0.699
	WBSS-SF	below mean	17/80 (21)	ref			21/79 (27)	ref		
		above mean	23/51 (45)	0.083	1.9 (0.8–4.8)	0.172	8/50 (16)	0.117	0.6 (0.2-2.0)	0.432
	Maternity leave†	< 3 months	4/27 (15)	ref			9/26 (35)	ref		
		≥ 3 months	36/103 (35)	0.396			20/103 (19)	0.102		
	Felt maternity leave was long enough	no	14/40 (35)	ref			12/38 (32)	ref		
		yes	25/90 (28)	0.539			17/90 (19)	0.079	0.3 (0.1–1.1)	0.063
	Expressed breastmilk at work	no	14/43 (33)	ref			**15/41 (37)**	ref		
		yes	26/88 (30)	0.497			**14/88 (16)**	**0.035**	0.7 (0.3-2.0)	0.509
	Distance from child	far	17/46 (37)	ref			16/46 (33)	ref		
		daycare/close^#^	22/84 (26)	0.421			14/82 (17)	0.053	**0.3 (0.1–0.8)**	**0.024**
Mother-infant dyad	Birth order of the last child	1	15/44 (34)	ref			6/42 (14)	ref		
		2	22/70 (31)	0.915	0.7 (0.2–2.8)	0.426	17/70 (24)	0.172	3.0 (0.8–11.5)	0.111
		≥ 3	3/17 (18)	0.159	0.3 (0.1–1.6)	0.161	6/17 (35)	0.148	**8.3 (1.4–48.8)**	**0.020**
	Breast rejection	no	27/92 (29)	ref			16/90 (18)	ref		
		yes	13/39 (33)	0.408			13/39 (33)	0.181	2.6 (0.9–7.4)	0.082
	Experience of breastfeeding	positive	**34/120 (28)**	**ref**			28/119 (24)	ref		
		neg/neutral	**6/8 (75)**	**0.005**	**9.2 (1.2–71.9)**	**0.034**	1/8 (13)	0.266		
Year of birth	Before 2018		30/96 (31)	ref			**26/94 (28)**	**ref**		
	2018–2021		10/35 (29)	0.533			**3/35 (9)**	**0.021**	**0.2 (0.0-0.8)**	**0.027**

All results in bold are those that are statistically significant (p-value < 0.05)

^#^ “Close” was defined as “close enough to visit the baby during the work day (e.g. at lunch break)”. † Omitted from multivariable regression because colinear with site. Abbreviations: BF breastfeeding, CBO community based organization, TH Thai Hospital, WBSS-SF Workplace Breastfeeding Support Scale– short form. Neg negative

* adjusted for work site, WBSS-SF, birth order, and experience of breastfeeding

** adjusted for work site, WBSS-SF, perception of adequate maternity leave, breast milk expression at work, distance from child, birth order, breast rejection after bottle feeding, and year of birth

Almost a quarter (39/169, 23%) of women reported the reason they stopped breastfeeding was work. (Tables 3 and 5). Multivariable regression found a lower risk of stopping breastfeeding due to work challenges among women who could see their babies during the work day (aOR 0.3, 95%CI 0.1–0.8, p 0.024), or whose most recent baby was born in the most recent year quartile (2018–2021, aOR 0.2, 95%CI 0.0-0.8, p 0.027), and a higher risk for women with two or more children before the most recent baby (aOR 8.3 compared with firstborn, 95%CI 1.4–48.8, p 0.020).

#### Caring for breastfeeding patients

The theme of the impact of personal breastfeeding experience on ability to care for breastfeeding dyads was also explored in the surveys through self-reported confidence caring for breastfeeding patients, congruence of advice given to patients about breastfeeding duration with WHO guidelines, and breastfeeding knowledge. Details are found in Table 6 and Additional File 5.

**Table 6:** Characteristics associated with improved confidence caring for breastfeeding dyads and with giving correct advice about recommended duration of breastfeeding. The results of univariable and multivariable logistic regression analysis of relationships between work site and personal factors and confidence and correct advice when caring for breastfeeding dyads are presented

		Confidence caring for breastfeeding dyads* *n* = 281				Breastfeeding advice consistent with WHO** *n* = 281			
**Characteristic**		proportion (%)	p	aOR (95%CI)	p	proportion (%)	p	aOR (95%CI)	p
Met both WHO goals	no	**108/235 (46)**				113/236 (48)	ref		
	yes	**21/30 (70)**	**0.016**	**2.6 (1.1–6.4)**	**0.033**	17/30 (57)	0.243	1.5 (0.7–3.4)	0.299
Low BF prevalence, low support (TH2)		31/61 (51)	ref	ref		45/68 (66)	ref		
Low BF prevalence, high support (TH1)		38/82 (46)	0.742	1.2 (0.6–2.8)	0.605	**27/59 (46)**	**0.022**	**0.4 (0.2–0.8)**	**0.009**
High BF prevalence, low support (CBO2)		32/68 (47)	0.930	1.4 (0.6–3.1)	0.382	**29/82 (35)**	**< 0.001**	0.5 (0.2–1.5)	0.207
High BF prevalence, high support (CBO1)		29/58 (50)	0.670	2.1 (0.9–4.9)	0.088	32/61 (52)	0.114	1.5 (0.5–4.6)	0.517
Age (years)^#^	< 30	29/72 (39)		**ref**		34/73 (47)	0.625		
	≥ 30	102/197 (52)	0.062	**1.9 (1.1–3.6)**	**0.032**	99/197 (50)	ref		
Occupation	Nurse or MW	**76/124 (61)**	**< 0.001**	**2.6 (1.5–4.7)**	**0.001**	67/125 (54)	0.241		
	Other	**54/145 (37)**		**ref**		66/145 (46)	ref		
Training	informal	72/155 (46)				**65/155 (42)**	**ref**		
	formal	58/114 (51)	0.473			**68/115 (59)**	**0.037**	2.1 (0.8–5.5)	0.139
Number of BF patients in the past 3 months	≤ 10	**25/72 (35)**		ref		**26/72 (36)**	**ref**	**ref**	
	> 10	**105/197 (53)**	**0.008**	1.6 (0.9–2.9)	0.145	**107/198 (54)**	**0.006**	**2.1 (1.2–3.7)**	**0.014**
Number of patients with breastfeeding problems in the past 3 months	0	**11/55 (20)**		**ref**		25/55 (45)	ref		
	≥ 1	**119/214 (56)**	**< 0.001**	**3.3 (1.6–6.5)**	**0.001**	108/205 (50)	0.621		
Breastfeeding attitude quartile (1 is the least positive, 4 is the most positive)	1	27/66 (41)	ref	ref		20/66 (30)	ref		
	2	29/72 (40)	0.940	0.8 (0.4–1.6)	0.506	**35/72 (49)**	**0.029**	**2.3 (1.2–4.7)**	**0.025**
	3	32/66 (48)	0.382	1.3 (0.6–2.7)	0.520	**36/66 (55)**	**0.006**	**3.4 (1.6–7.1)**	**0.001**
	4	**42/64 (66)**	**0.005**	1.8 (0.7–4.2)	0.193	**42/65 (65)**	**0.001**	**2.6 (1.2-6.0)**	**0.021**

All results in bold are those that are statistically significant (p-value < 0.05)

Note: Duration of breastfeeding by self or partner, positive or negative breastfeeding experience, breastfeeding knowledge score, and high school education were not associated with confidence or advice consistent with WHO.

Abbreviations: BF breastfeeding, CBO community based organization, ref reference, TH Thai hospital, WHO World Health Organization

^#^ < 30 was the lowest quartile for overall staff age

* Adjusted for meeting WHO goals, work site, age, occupation, number of breastfeeding patients, number of patients with breastfeeding problems, breastfeeding attitude quartile, and also for sex and advice consistent with WHO recommendations (not significant)

** Adjusted for meeting WHO goals, work site, training, number of breastfeeding patients, breastfeeding attitude quartile, and also for confidence (not significant)

Personally meeting both WHO breastfeeding recommendations (aOR 2.6, 95%CI 1.1–6.4 p 0.033) was associated with high confidence in caring for breastfeeding dyads, as were older age (≥ 30 years, aOR 1.9, 95%CI 1.1–3.6, p 0.032), being a nurse or a midwife (either formally or informally trained, aOR 2.6, 95%CI 1.4–4.7, p 0.001), and caring for patients with breastfeeding problems (aOR 3.3, 95%CI 1.6–6.5, p 0.001).

Caring for pregnant or breastfeeding patients (aOR 2.1, 95%CI 1.2–3.7, p 0.001), and higher breastfeeding attitude scores (for the highest quartile compared to the lowest: aOR 2.6, 95%CI 1.2-6.0, p 0.021) were associated with correct breastfeeding duration advice. Notably, TH2 (low breastfeeding prevalence, low support) staff reported the most correct advice, significantly better than TH1 (low breastfeeding prevalence, high support, aOR 0.4 95%CI 0.2–0.8, p 0.009). Overall knowledge scores were low (Additional File 5), with a mean score of 34.7% (sd 15.5, Table 2). Higher scores were associated with formal Thai or Myanmar training (aOR 3.5, 95%CI 1.3–9.6, p 0.013), or working for the more supportive sites: TH1 (aOR 4.1, 95%CI 1.6–10.6, p 0.003) and CBO1 (aOR 3.2, 95%CI 1.0–10.0, p 0.050).

Sensitivity analysis of the impact of non-random sampling in TH1 is included as Additional File 6.

## Discussion

This study provides needed insight into how workplace drivers influence HCW breastfeeding practice, and in turn, how this influences HCW ability to care for breastfeeding patients. Both quantitative and qualitative data suggest that important gains in EBF-6 m among HCW can be attained through organizational support, particularly comprehensive support in multiple domains such as demonstrated in a resource-constrained setting at CBO1. Consistent with results from previous literature, qualitative results highlighted length of maternity leave and option to extend if needed [18, 35, 36]; respite from night duty [37]; access to clinical breastfeeding support to solve breastfeeding problems [38, 39]; and availability of child-friendly spaces at work as important components of desired support [13]. Breastfeeding outcomes, particularly EBF-6 m, were significantly better at CBO1 with 3 months’ leave, flexible accommodations for individual staff, and a functioning daycare facility than at CBO2 with 2 months’ maternity leave and no lactation facilities, despite very similar cultural influences. The decreased incidence of breastfeeding cessation due to work among healthcare workers who could see their infants during the work day across all sites is further evidence that well-designed, child-friendly spaces can be an effective intervention. Flexible policies were a double-edged sword, in some cases allowing needed accommodations, but in other cases leaving mothers without guaranteed protections.

Across the diverse facilities represented here, and in both the qualitative and quantitative data, healthcare workers who met WHO recommendations when breastfeeding their own children were more confident to care for breastfeeding dyads. The interviewees unanimously stated that this confidence followed (and did not pre-date) their breastfeeding experience. Healthcare worker confidence is an essential element of effective clinical care [40] and Cochrane meta-analyses have repeatedly found that professional support for breastfeeding dyads improves breastfeeding duration and exclusivity for patients [41]. Confidence needs to be paired with clinical competence. This study also sheds light on gaps in training about breastfeeding medicine, particularly in Tak province, Thailand. Pairing effective and practical breastfeeding education with support for personal breastfeeding in this kind of setting is likely to lead to health benefits in local communities [42].

As in other settings, work and low milk production were the two most commonly cited reasons for terminating breastfeeding [39, 43]. Work and low milk production are correlated and interventions that facilitate timely breast emptying and direct breastfeeding for working mothers are likely to decrease early cessation of breastfeeding from both causes. Skilled evaluation and care for breastfeeding problems, as suggested by Thai interview participants, is likely to have a significant impact on duration and exclusivity, especially in workplaces with less prevalent breastfeeding experience. The association between low milk production and negative breastfeeding experiences highlights the trauma these women can experience when breastfeeding promotion lacks sensitivity and practical support.

There were hopeful signs for the future: staff who set breastfeeding goals were more likely to EBF-6 m and to reach both WHO recommendations. Goal-setting has been suggested as a useful step to improve breastfeeding duration and exclusivity [44, 45], coupled with effective support to help parents achieve those goals in order to avoid increasing guilt and shame relating to infant feeding [46]. Mothers who had their most recent child after 2015 were more likely to meet both WHO recommendations and less likely to quit breastfeeding because of work, indicating overall progress in participating organizations. Since the completion of this study, CBO2 has extended maternity leave to 3 months and TH2 has opened a lactation clinic.

The WBSS performed poorly in this study, with the highest scores at TH2, which had the shortest breastfeeding durations and most negative qualitative descriptions of breastfeeding support. However, TH2 staff scored highly in positive attitudes towards breastfeeding and accurate advice to patients. The WBSS may be unable to detect the difference between theoretical breastfeeding support and practical support of breastfeeding mothers, especially in a culture where staff may be hesitant to criticize their superiors.

There were some limitations to the sample. With less than 5% of women taking more than 3 months’ leave, there was not a sufficient range in lengths of maternity leave to assess the quantitative impact of longer leave. By surveying women at work, the voices of women who left work in order to stay home with their children were missed. The lower proportion of mothers among staff at the less supportive sites suggests that one cost of unsupportive maternity policies is loss of experienced staff. The 84 item survey did not include some relevant questions including skin to skin time at birth (generally low in this setting), partner support, or concerns about the impact of breastfeeding on breast shape. Breastfeeding rates were lower at organizations with higher salaries, implying that formula feeding might be a privileged option not available to all, but data on individual salaries was not collected to investigate this relationship more. This study also does not address the logistical and financial difficulties faced by organizations, especially in rural locations, to accommodate breastfeeding supportive policies. Knowledge scores were low. However, the tool used here, which was originally developed for physicians in Australia [15] may be poorly suited to the non-physician participants. Conducting surveys across multiple languages allowed for broad sampling, but subtle differences in tone of questions in different languages may have influenced results. An extensive translation verification process minimized this risk.

The greatest strength of this study was its mixed-methods approach, which has been rarely applied to this complex question in low- and middle-income settings. During analysis, results of the qualitative data informed the conceptual framework used to build the logistic regression model, while the quantitative data highlighted the parts of the interviews that were most generalizable. Feedback sessions with participating organizations served to triangulate the data and strengthen the validity of the final results. A further strength was the use of previously validated questionnaires [15]. Studies in low- and middle-income countries prior to this work have been limited by underpowering or limited analysis [47–49], often using mail surveys with < 50% response rates or convenience sampling. The predominantly probabilistic sampling approach in this study allowed for a stronger analysis of associations. Though bias may have been introduced by convenience sampling in the TH1 data, the sensitivity analysis (Additional File 6) was reassuring.

Though local influences on breastfeeding behavior abound, the multi-facility and multi-cultural contexts of this study suggest that the results can be generalized beyond the immediate setting. Congruence with work from other settings further supports generalizability beyond Southeast Asia.

## Conclusions

Workplace support and breastfeeding goal setting were associated with longer breastfeeding duration and exclusivity for healthcare workers in Northern Thailand. These healthcare workers had greater confidence caring for breastfeeding dyads after breastfeeding their own children. Mothers highly valued being able to see their infants during the workday and this was associated with a lower risk of abandoning breastfeeding due to pressure from work.

### Electronic supplementary material

Below is the link to the electronic supplementary material.

Supplementary Material 1: Supplementary material includes the interview guide, quantitative survey, study recruitment flow, additional results and sensitivity analysis

## Data Availability

Data is available for further analysis upon reasonable request. Due to sensitive documentation status of some of the participants in this study, the data is retained securely by the investigators rather than in a public repository.
