# Supplementary material for: Determinants of health care worker breastfeeding experience and practices and their association with provision of care for breastfeeding mothers: a mixed-methods study from Northern Thailand
Source: Int Breastfeed J. 2024 Jan 25;19:8. doi: 10.1186/s13006-024-00613-4 (PMC10809554; doi:10.1186/s13006-024-00613-4)
Supplement: Supplementary file 1 — Supplementary Material 1: Supplementary material includes the interview guide, quantitative survey, study recruitment flow, additional results and sensitivity analysis [file 13006_2024_613_MOESM1_ESM.docx]

**Additional File 1: Semi-structured interview guide**

*Ice breaker: How many children do you have? How old are they? Where were they born? (Casual, just to start the conversation.)*

What was your experience working in the first year after having your baby?

*Prompts (if needed)*

- How was that experience – was it easy or hard?
- What problems did you have?
- What may have made this time easier for you than for other people you worked with who had infants?
- Have you had experience working just after delivery AND having a delivery but not having to work? What was the difference?

*If the participant does not speak about feeding practices spontaneously within 5-10 minutes:*

Before your delivery, what was your plan to feed your baby?

- Is that what happened in real life? *Or* Did your plan change? Why or why not?
- What helped you follow your plan? What prevented you from following your plan?
- How do you feel about how your baby was fed?

Do you think that there are ways that an employing organization can help working mothers feed their babies how they want to? What ideas do you have?

If your sister, close friend or coworker is thinking about starting a family, what advice would you give her?

- How would you advise her about infant feeding?
- Would you ever recommend delaying childbearing until after leaving this job? Why or why not?
- Would you ever recommend quitting the job to have kids? Why or why not?

For your job, do you take care of mothers who are breastfeeding, or their babies?

- If a patient has a breastfeeding problem (eg. not making enough milk for the baby), Do you feel you are able to help her? Why or why not?
  - Do you think breastfeeding problems can always be solved? Why or why not? If not always, how often can they be solved?
- How do you feel about this work? Do you enjoy working with these moms? Do you dread it? Why?
  - Do your own breastfeeding experiences help you when taking care of these women? Why or why not?
  - Do you share your own stories or challenges?

**Additional File 2: Survey**

**Modified Australian breastfeeding knowledge and attitude questionnaire adapted for the Thailand Myanmar Border**

**Thank you for filling out this questionnaire. The results will be kept confidential.**

Date |__|__| |__|__|__| |__|__|__|__|

**First, we would like to ask you some questions about yourself.**

1. **What is your gender?** ^a^ Male ^b^ Female

1. **How old are you?** (years)

1. **In what country were you born?** ^a^ Thailand ^b^ Myanmar ^c^ Other
2. **Were you born in a refugee camp? ^a^** Yes **^b^** No
3. **How long have you worked as a nurse/midwife/medic/doctor (total time) etc?**

|___| years |___| months

1. **Where have you worked?**
2. **Where did you complete your doctor/ nurse/midwife/medic training?** (Can check more than 1)

^a^ Thailand formal ^b^ Myanmar formal ^c^ Thailand NGO

^d^ Myanmar NGO ^e^ Refugee camp ^f^ Other _________________

1. **Before your doctor/medic/midwife/nurse training, what grade did you get to in school?**

^a^ Some primary school ^b^ Finished primary school

^c^ Some high school ^d^ Finished high school

^e^ Some University ^f^ Finished University

^g^ Post-university (Master’s, Doctorate)

1. **What is your current job?**

^a^ Medic ^b^ Midwife ^c^ Nurse

^d^ Health Worker/Nurse Aid ^e^ Ultrasound ^f^ Lab

^g^ Cook ^h^ Cleaner ^i^  Doctor

^j^ Other:___________________

1. **In which areas have you worked (not as student)? (can choose more than 1)**

^a^ Reproductive Health (RH) Outpatient department (OPD) **or** Antenatal Care (ANC)

^b^ Any Adult Department

^c^ RH inpatient (IPD) **or** Delivery room **or** Postpartum inpatient unit

^d^ Neonatal IPD **or** NICU **or** Special Care Baby Unit (SCBU)

^e^ Neonatal **or** Pediatric OPD

^f^ Pediatric IPD

^g^ Lab

^h^ Kitchen

^i^ Other: __________________

**To what extent do you agree or disagree with the following statements about breast milk and breastfeeding? Please mark the appropriate response.**

|  | Strongly disagree | Disagree | Neither agree or disagree | Agree | Strongly agree |
| --- | --- | --- | --- | --- | --- |
| 1. Infant formula is more easily digested than breast milk. |  |  |  |  |  |
| 1. Breast milk is the ideal food for babies. |  |  |  |  |  |
| 1. Breastfeeding increases mother-infant bonding. |  |  |  |  |  |
| 1. Breastfeeding provides health benefits for infants that cannot be provided by infant formula. |  |  |  |  |  |
| 1. Breastfeeding is incompatible with working outside the home. |  |  |  |  |  |
| 1. Fathers feel left out if a mother breastfeeds. |  |  |  |  |  |
| 1. Infant formula is as healthy for an infant as breast milk. |  |  |  |  |  |
| 1. Formula feeding is the better choice if the mother plans to go out to work. |  |  |  |  |  |
| 1. The benefits of breast milk last only as long as the baby is breastfed. |  |  |  |  |  |
| 1. Formula feeding is more reliable because you can calculate the exact quantity of milk the baby is getting. |  |  |  |  |  |
| 1. Current infant formulas are nutritionally equivalent to breast milk. |  |  |  |  |  |
| 1. Women should not breastfeed in public places such as restaurants. |  |  |  |  |  |

1. **Did your mother breastfeed you?**

**^a^** Yes **^b^** No **^c^** Don’t know

1. **Do you have children of your own?**

^a^ Yes: How many? ______ ^b^ No (go to question 61)

1. **Did you ever quit your job so you could stay home with your young children?**

**^a^** Yes **^b^** No

1. **Were any of your children breastfed?**

**^a^** Yes **^b^** No , not at all (go to question 29)

1. **What is the TOTAL length of the personal breastfeeding experience of you or your partner for your children?**

*For example: if your wife breastfed your first child for 18 months and your second child for 2 years, the total = 3.5 years*

^a^ Less than 2 weeks ^b^ 2 – 12 weeks

^c^ 13 - 26 weeks (3-6 months) ^d^ 27 weeks (>6 mos) – 52 weeks (1 year)

^e^ > 1 year to 2 years ^f^ > 2 years to 5 years

^g^ > 5 years

1. **Overall, how was the personal breastfeeding experience of you or your partner?**

^a^ Positive ^b^ Negative

^c^ Neutral (not positive or negative) ^d^ Don’t know

**Mother Breastfeeding Experience Questionnaire**

Mothers, please answer these questions about your last baby.

1. Child # _____
2. What kind of delivery? ^a^ Normal ^b^ Cesarean Section ^c^ Vacuum/Forceps

1. In what year was your child born? __________
2. Where was this baby born?:

^a^ MTC ^b^ SMRU ________________

^c^ IRC Clinic ^d^ Home

^e^ Thai Hospital ___________________ ^f^ Myanmar Hospital _________________

^g^ Other: ________________________

1. Did you have a plan or goal to breastfeed this child?

**^a^** No **^b^** Yes but no planned time

**^c^** Yes: I planned to give **only** breastmilk (no water, powder milk or food) ______ mos,

I planned to give breast milk to **total** ______ mos

1. How long did you breastfeed this child?

I fed **only** breastmilk (no water, powder milk or food) ______ mos,  Still feeding only breastmilk

I breastfed for a total of **total** ______ mos  Still breastfeeding

1. When did you first give your baby:

Water ^a^ at________ mos ^b^ Never/Not yet ^c^ Don’t remember

Powder milk ^a^ at________ mos ^b^ Never/Not yet ^c^ Don’t remember

^d^ Small amount of powder milk only in the first month of life

Other milk products ^a^ at________ mos ^b^ Never/Not yet ^c^ Don’t remember

Food/ rice ^a^ at________ mos ^b^ Never/Not yet ^c^ Don’t remember

1. Why did you stop breastfeeding (pick the most important reason)?

^a^ N/A (never breastfed) ^b^ N/A (still breastfeeding)

^c^ Mother feels the baby no longer needs breastmilk ^d^ Not enough breast milk

^e^ Difficult to breastfeed while working ^f^ Pregnant again

^g^ Baby stopped by self ^h^ Mother medical problem

^i^ Baby medical problem ^j^ Baby far away with family

^k^ Other: **___________________________________________**

1. Did you work while this baby was less than 1 year old? **^a^** Yes **^b^** No (go to question 61)
2. *If yes to 37*, where did you work?

^a^ MTC ^b^ SMRU _______________________

^c^ Mae Ramat Hospital ^d^ Sarapee hospital

^e^ Other: _______________________

1. How long was your maternity leave? _______ mos Don’t remember
2. How many months after delivery were you put on night duty? ______ mos Don’t remember N/A

**If you worked at MTC, SMRU, Mae Ramat Hospital or Sarapee Hospital while your last baby was less than one year old, please answer these questions**

|  | Yes | No | Don’t remember |
| --- | --- | --- | --- |
| 1. Baby stayed close, and I could visit my baby during the work day. |  |  |  |
| 1. I used on-site day care at work. |  |  |  |
| 1. Baby stayed too far to visit during the work day, but I saw my baby when not on duty. |  |  |  |
| 1. I sent my baby to stay with relatives. I could not see my baby daily. |  |  |  |
| 1. I expressed breast milk for the baby to take while I was at work. |  |  |  |
| 1. I had difficulty finding someone to care for the baby near the clinic. |  |  |  |
| 1. My maternity leave was long enough to get a good start on breastfeeding. |  |  |  |
| 1. I felt I had to go back to night duty too soon after delivery. ( N/A) |  |  |  |
| 1. I had difficulty getting this baby to accept the breast after getting bottle fed. ( N/A) |  |  |  |

| Workplace Breastfeeding Support Scale | Strongly disagree | Disagree | Neither agree or disagree | Agree | Strongly agree | Don’t remember/ Don’t know |
| --- | --- | --- | --- | --- | --- | --- |
| 1. My workplace has a refrigerator that I can use to store my milk. |  |  |  |  |  |  |
| 1. Breastfeeding is common in my workplace. |  |  |  |  |  |  |
| 1. I have a breastfeeding-supportive supervisor. |  |  |  |  |  |  |
| 1. My workplace has an on-site daycare. |  |  |  |  |  |  |
| 1. In my workplace, there is a designated space (nursing room) to nurse my baby or pump breast milk. |  |  |  |  |  |  |
| 1. My workplace has a breast pump for nursing mothers to use. |  |  |  |  |  |  |
| 1. My coworkers agree that breastfeeding is better for a baby’s health than formula feeding. |  |  |  |  |  |  |

**If you did not breastfeed or express breast milk at work, go to question 61.**

**If you breastfed or expressed breast milk at work, please answer questions 57 to 60.**

| Workplace Breastfeeding Support Scale | Strongly disagree | Disagree | Neither agree or disagree | Agree | Strongly agree | Don’t remember/ Don’t know |
| --- | --- | --- | --- | --- | --- | --- |
| 1. I feel comfortable taking several breaks during work hours to pump breast milk. |  |  |  |  |  |  |
| 1. I have supportive coworkers who cover for me when I need to express my milk. |  |  |  |  |  |  |
| 1. My coworkers do not make fun of me when I sometimes leak milk through my clothes. |  |  |  |  |  |  |
| 1. My coworkers listen to me talk about my breastfeeding experience. |  |  |  |  |  |  |

1. **In the past 3 months, how many patients have you seen in TOTAL who were:**
   1. **pregnant,**
   2. **mothers of infants under 12 months of age**

**or**

- 1. **infants under 12 months of age?**

**(please mark the most appropriate response)**

^a^ No patients ^b^ 1-10 ^c^ 11-20 ^d^ 21-30 ^e^ 31-50 ^f^ >50

1. **In the past 3 months how many patients have you seen with a problem related to breastfeeding? (please mark the most appropriate response)**

^a^ No patients ^b^ 1-5 ^c^ 6-10 ^d^ 11-15 ^e^ 16-20 ^f^ >20

**If your response to Question 61 and 62 was “no patients” you are finished with the survey.**

**If you see these patients, please continue with Question 63 relating to your confidence and knowledge about breastfeeding and breast milk.**

1. **How confident would you say you are with your ability to assist women who present with breastfeeding problems? (please mark one response)**

| Not very confident  1 | 2 | 3 | 4 | Very confident  5 |
| --- | --- | --- | --- | --- |

1. **Around what age do you recommend solids be introduced to a breastfed infant (nearest value)?**

^a^ 3 months ^b^ 6 months ^c^ 9 months ^d^ 12 months

     

^e^ Other­­­­­­­­­­­­­­­­­­­­­­­­­­­­­­­

1. **For how long do you recommend to a mother that she continue to breastfeed her infant? (Choose one.)**

^a^ No longer than 6 months  ^b^ Until teeth erupt

^c^ Around 9 months ^d^ No longer than 12 months

^e^ Between 12 and 24 months ^f^ 2 years and beyond

     

^g^ For as long as the mother and baby wish ^h^ Other

**Are the following statements correct or incorrect? Please mark the appropriate response. If you are unable to give a response to a statement, mark the last response column headed “don’t know”.**

|  | Correct | Incorrect | Don’t know |
| --- | --- | --- | --- |
| 1. Breastfed infants require extra water in hot weather. |  |  |  |
| 1. It is expected that breastfed infants will regain their birth-weight by two weeks of age. |  |  |  |
| 1. It is normal for an adequately breastfed 2 -week old infant to only pass a bowel motion every 3 days or so. |  |  |  |
| 1. Women who have breastfed have a lower incidence of premenopausal breast cancer. |  |  |  |
| 1. Amoxycillin is the drug of choice to treat mastitis in a woman 3 months postpartum. |  |  |  |
| 1. All women with cracked nipples should express their milk and rest the nipples for 24 hrs. |  |  |  |
| 1. High maternal prolactin levels are essential for the initiation of lactation. |  |  |  |
| 1. Introducing complementary feeds (water or formula) interferes with the establishment of breastfeeding. |  |  |  |
| 1. Antenatal nipple preparation prevents nipple soreness in the first week postpartum. |  |  |  |
| 1. A nipple shield should be used if there are any problems with the infant attaching to the breast. |  |  |  |
| 1. Breastfeeding is contraindicated for women with Hepatitis B. |  |  |  |
| 1. The nutritional content of breast milk changes throughout a breastfeed. |  |  |  |
| 1. The most common cause of cracked nipples is poor positioning and attachment of the infant at the breast. |  |  |  |
| 1. In most cases a breastfeeding mother must temporarily wean her baby while she is taking prescription medications. |  |  |  |
| 1. Growth of breastfed infants differs from that of formula fed infants. |  |  |  |
| 1. In general, the most appropriate advice to give a woman with a low milk supply is to increase the frequency of breastfeeds. |  |  |  |
| 1. A ‘top-up’ bottle after each breastfeed is the best way to manage an infant who is not gaining weight adequately. |  |  |  |
| 1. Breastfeeding reduces the incidence of gastroenteritis in the infant. |  |  |  |
| 1. Only feeding from one breast at each feed is a management option for a woman with an oversupply of breast milk. |  |  |  |

**Thank you very much for taking the time to complete the questionnaire.**

**Please check that you have answered all the questions.**

**Additional File 3: Inclusion flow**


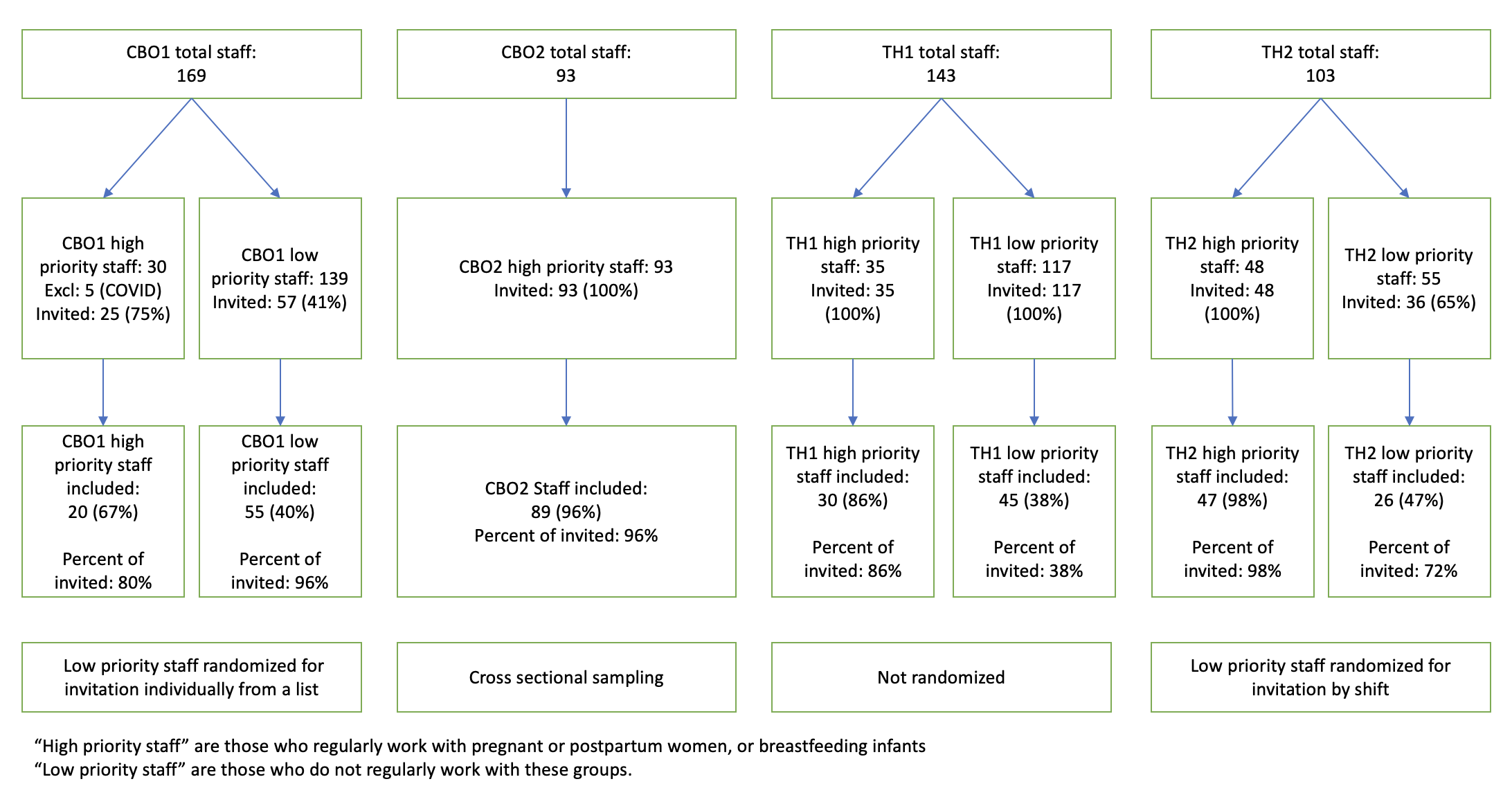


At CBO2, all staff care for postpartum women or infants, and were invited. CBO1 health information team used computer randomization to determine invitations for staff working in low-priority departments. At TH2, staff were randomized by shift (e.g. morning shift) after confirming that all staff rotate regularly through the shifts. At TH1 randomization was not possible and convenience sampling was used (see *Bias* section.)

**Additional File 4: Characteristics associated with staff meeting both WHO exclusive and total breastfeeding goals**

|  | | | **Exclusive breastfeeding to 6 months & total breastfeeding 2 years** | | | |
| --- | --- | --- | --- | --- | --- | --- |
|  |  |  | univariable (clustered) | | Multivariable (N=129) | |
| **Characteristic** | | | proportion (%) | p | aOR (95%CI) | p |
| Work site | Low BF culture, low support (TH2) | | 2/35 (6) | ref | - |  |
|  | Low BF culture, high support (TH1) | | 5/37 (14) | 0.278 | 3.8 (0.6-23.4) | 0.155 |
|  | High BF culture, low support (CBO2) | | 5/24 (21) | 0.097 | 2.3 (0.3-17.1) | 0.853 |
|  | High BF culture, high support (CBO1) | | **15/33 (45)** | **0.001** | **8.3 (1.4-48.4)** | **0.018** |
| Mother/ infant dyad | Place of birth^#^ | clinic/home | **14/31 (45)** | **ref** |  |  |
|  |  | hospital | **13/98 (13)** | **0.020** |  |  |
|  | Set a goal | no | **2/40 (5)** | **ref** |  |  |
|  |  | yes | **25/89 (28)** | **0.023** | **5.5 (1.1-27.3)** | **0.036** |
|  | Baby rejected the breast after bottle | no | **22/85 (26)** | ref |  |  |
|  |  | yes | **5/39 (13)** | 0.112 | 0.3 (0.1-1.3) | 0.105 |
| Year of Birth | 1990-2015 | | **8/68 (12)** | **ref** |  |  |
|  | 2016-2021 | | **19/61 (31)** | **0.014** | **3.3 (1.2-9.5)** | **0.030** |

* Adjusted for work site, goal setting, breast rejection, and year of birth as well as high school education and reason for weaning (not significant)

^#^ “Clinic/home” group was predominantly clinic-based births. This variable was excluded from multivariable analysis because of collinearity with work site.

Abbreviations: WHO World Health Organization, BF breastfeeding

**Additional File 5: Characteristics associated with breastfeeding knowledge scores about the 50^th^ centile**

|  |  | **Knowledge breastfeeding dyads** | | | |
| --- | --- | --- | --- | --- | --- |
|  |  | univariable (clustered) | | Multivariable (N=226)* | |
| **Characteristic** | | proportion (%) | p | aOR (95%CI) | p |
| Low BF culture, low support (TH2) | | 43/67 (64) | ref | ref |  |
| Low BF culture, high support (TH1) | | **48/57 (84)** | **0.014** | **4.1 (1.6-10.6)** | **0.003** |
| High BF culture, low support (CBO2) | | 40/82 (48) | 0.061 | 1.8 (0.6-5.3) | 0.287 |
| High BF culture, high support (CBO1) | | 36/59 (61) | 0.714 | 3.2 (1.0-10.0) | 0.050 |
| Sex | female | 149/227 (66) | 0.054 | 1.7 (0.8-3.7) | 0.139 |
|  | male | 18/38 (47) |  | ref |  |
| Training | informal | **81/152 (53)** |  | **ref** |  |
|  | formal | **86/113 (76)** | **0.003** | **3.5 (1.3-9.6)** | **0.013** |
| Number of patients with BF problems in the past 3 months | 0 | 28/53 (53) |  | ref |  |
|  | ≥1 | 138/211 (65) | 0.168 | 1.5 (0.8-2.8) | 0.159 |
| Meet both WHO goals | no | 143/231 (62) |  |  |  |
|  | yes | 22/30 (73) | 0.193 | 1.9 (0.8-4.8) | 0.155 |

Note: Age, length of work in healthcare, duration of breastfeeding by self or spouse, occupation (nurse or midwife vs others), high school education, number of breastfeeding patients seen in the past 3 months, confidence and consistency of advice with WHO recommendations were all not associated with knowledge scores.

* Adjusted for work site, sex, training, number patients with breastfeeding problems, and meeting WHO recommendations.

Abbreviations: WHO World Health Organization, BF breastfeeding

**Additional File 6**

*Sensitivity analysis*

Sensitivity analysis was done on all regression analyses, omitting the TH1 data. Loss of statistical significance for some variables was expected due to decreased sample size, however the overall results were very similar. The associations in the analysis of exclusive breastfeeding with the exception of expressing breast milk at work, which increased in magnitude and approached significance (aOR 4.1, 95%CI 0.9-17.8, p 0.064), and low education, which barely lost significance (aOR 4.7, 95%CI 1.0-22.2, p 0.53). The direction and magnitude of the association between total breastfeeding duration and low milk production (aOR 0.2, 95%CI 0.0-1.2, p 0.079), and weaning due to work (aOR 0.3, 95%CI 0.1-1.3, p 0.110) remained the same but lost significance. In the analysis of meeting both WHO targets, setting a goal barely lost significance (aOR 8.4, 95%CI 0.9-74.5, p 0.56).

The relationship between weaning due to low milk production and negative breastfeeding experience (aOR 6.2, 95%CI 0.6-62.4, p 0.122) or work at CBO2 (aOR 0.2, 95%CI 0.0-1.5, p 0.121) lost significance but had similar effect size and direction. Without the TH1 data, higher WBSS-SF score was associated with a higher risk of weaning due to insufficient milk production (aOR 3.8, 95%CI 1.0-14.2, p 0.044), likely partially due to high WBSS scores at TH2, where low milk production was common. In the wean due to work analysis, two covariates lost significance: high birth order (aOR 3.6, 95%CI 0.4-33.2, p 0.254), and birth after 2017 (aOR 0.30, 95%CI 0.1-1.4, p 0.130), while the magnitude and direction of the effect remained. The variable associations with confidence were unchanged from the whole cohort, and, in the analysis of breastfeeding advice, only the association with caring for breastfeeding patients lost significance (aOR 1.8, 95%CI 1.0-3.5, p 0.064). After excluding TH1 participants, the association between higher breastfeeding knowledge scores and training in the formal system lost significance (aOR 2.4, 95%CI 0.8-7.9, p 0.135).
